# The Effect of Doulas on Maternal and Birth Outcomes: A Scoping Review

**DOI:** 10.7759/cureus.39451

**Published:** 2023-05-24

**Authors:** Alexandria Sobczak, Lauren Taylor, Sydney Solomon, Jodi Ho, Scotland Kemper, Brandon Phillips, Kailey Jacobson, Courteney Castellano, Ashley Ring, Brianna Castellano, Robin J Jacobs

**Affiliations:** 1 Medicine, Nova Southeastern University Dr. Kiran C. Patel College of Osteopathic Medicine, Fort Lauderdale, USA

**Keywords:** childbirth satisfaction, delivery and labor, doula, health disparity, labor and birth, treatment disparity

## Abstract

A source of support during birth could be the solution to negative outcomes for the mother and her baby. To improve the birthing experience and increase positive birthing outcomes, sources of support during pregnancy should be evaluated and understood. The goal of this review was to synthesize the existing literature on how doulas might improve birth outcomes. This scoping review also aimed to shed light on the positive impact emotional support during childbirth can have on the health and well-being of mother and child. PubMed and EBSCOhost were used to identify articles using the search words with Boolean operators “doulas” AND “labor support” AND “birth outcomes” AND “pregnancy” AND “effects during labor.” The eligibility criteria for article selection included primary studies investigating how doulas contributed to birth outcomes. The studies in this review indicated that doula guidance in perinatal care was associated with positive delivery outcomes including reduced cesarean sections, premature deliveries, and length of labor. Moreover, the emotional support provided by doulas was seen to reduce anxiety and stress. Doula support, specifically in low-income women, was shown to improve breastfeeding success, with quicker lactogenesis and continued breastfeeding weeks after childbirth. Doulas can be a great resource for birthing mothers, and consideration should be given to using them more, as they may have a positive impact on the well-being of the mother and child. This study raised questions about the accessibility of doulas and how they may help mitigate health disparities among women from different socioeconomic levels.

## Introduction and background

Although parturition is still a relatively consistent process among women, support during labor can have a multitude of forms; partners, doulas, and healthcare providers are just a few types of support that women can have during childbirth. When a mother does not have support during childbirth, there can be negative results for the child and the mother. Some of the undesirable outcomes for the baby can be higher rates of low birth weight (LBW) or even preterm birth (PTB) [1]. These results can cause serious health issues as the infant’s life progresses [2]. As for the mother, well-being is always a concern during childbirth, especially in the United States where the maternal mortality rate, 23.8 deaths per 100,000 live births, is relatively high [3]. Statistics for mothers of lower socioeconomic status (SES) are proven to be even worse [4]. A source of support during parturition could be the solution to many adverse outcomes for the mother and the baby. To increase the viability of births and the survival of mothers, sources of support during pregnancy should be evaluated and understood.

Enduring a stressful birth experience is not uncommon [5] and can have severe long-lasting consequences for both mother and child, and individuals with low social support are especially at risk [4]. Socioeconomic status (SES) is a reliable method for predicting health disparities among a population [1]. Among other indicators of SES, neighborhood SES, such as those of minorities or stigmatized populations, is associated with adverse birth outcomes including preterm birth (PTB) or low birth weight (LBW) [1,6]. Additionally, maternal stress is associated with adverse birth outcomes [7], a significant public health issue with PTB and LBW leading to stunted growth and chronic conditions (e.g., obesity); these conditions are considered the largest risk factors for infant mortality [6].

According to Sentell et al. [8], non-English speakers had significantly more delivery complications than English speakers. Researchers investigating traumatic birth experiences found four common themes in the literature: prioritizing the care provider’s agenda, disregarding embodied knowledge, lies and threats, and violation [9]. Care providers can intimidate mothers by threatening the well-being of the baby. Mothers without support or who speak a different language than the provider are especially vulnerable in these cases.

There are many aspects of social support that need to be fulfilled during labor and delivery regarding both the mother and the child. Companionship and partner engagement during labor serve as emotional aspects of social support. By accompanying the mother, it gives them a sense of reassurance and confidence to manage anxiety and the stress that comes with labor [10]. According to Shah et al. [11], there was a reduction in pregnancy loss and LBW pregnancies just with the presence of a partner. Alongside emotional aid, there is also tangible support. This would include having a sterile environment for delivery, the skill and knowledge of the nurses and the physician, and the possible use of an epidural during delivery. For instance, the use of an epidural would provide practical support by supplying pain relief, decreasing the risk of acidosis, and reducing the risk of naloxone administration during parturition [12]. Tangible support could also include the use of interpreters if there is a language barrier present. Language disparities were associated with an increased number of cesarean births and a higher risk of obstetric trauma [8].

There is also the informational aspect of support, which includes messages being relayed to the mother such as facts or feedback during the birth. This assists with another aspect of social support known as appraisal support. Appraisal support differs from emotional support as it aids more in the self-reflection and evaluation of oneself during a situation rather than just general feelings one may experience [13]. Low self-efficacy during pregnancy was highly correlated to fears about birth, the fear of one’s ability to get through the birthing process, and fears of fetal safety [14]. Therefore, feelings of self-doubt and helplessness need to be decreased while feelings of control from the mother need to be supported.

Emotional support, tangible support, informational support, and appraisal support need to be equally sustained and looked at to fully analyze the impact of each labor support modality. In an interview conducted by Kazal et al. [15], mothers explained that the stress and anxieties related to “social networks and newborn care were of the highest importance.” While all the diverse types of support are present, they stressed the need to bridge the gap between all aspects of support as the worries came from receiving all aspects of support from different sources [15]. Hence, it was decided that the focus of this study is to be on doulas. With the use of doulas, the knowledge gaps that the mother may have on medical procedures, emotional stress of birthing, empathy, reassurance, nonjudgmental communication, and overall birth satisfaction may be fulfilled all at the same time and coincide together in a more comprehensive way.

Doulas can serve as advocates for patient autonomy, mitigating health disparities in groups at risk due to racial and socioeconomic stigmas via their roles as intermediaries between pregnant women and healthcare staff [16]. Programs matching vulnerable pregnant patients with community doulas look to pair experienced support personnel with a pregnant patient throughout the entirety of the pregnancy. In the antenatal and postnatal phases of pregnancy, doulas function both as supportive home visitors and maternal educators, but their role dramatically expands during delivery [17]. During parturition, doulas serve as nonmedical personnel focused on providing continuous support, encouragement, and guidance to patients through the rigors of labor and delivery. As advocates and intermediaries between physician and patient, doulas educate women on what to expect during labor, allow for improved communication for the patient, and establish birthing plans [18].

Doula support also extends to the physical processes of parturition, through which doulas help mothers cope with pain through positional changes, stretches, and massage techniques. With the sole role of supporting the patient’s physical, emotional, and social needs, doulas may help reduce peripartum anxiety by reassuring and empowering laboring mothers [19]. While it is noted that continuous support by external factors such as doulas may increase positive feelings about their labor experience, correlation to postpartum psychology is difficult given various social factors influencing both parents and children. Broadly, most studies have found correlations between continuous support and positive maternal outlooks, widely seen as beneficial compared to without these resources [20]. However, this focus presents unique questions about triggers of postpartum depression (PPD), the decision to have another child or stop having children altogether when the resources of a doula were used. A greater focus on how doulas influence maternal perceptions and outcomes should be analyzed through both the unique training of doulas from different cultures and their effectiveness in fostering beneficial relationships with mothers of lesser-studied populations, namely, those living under low SES [21]. Hence, this scoping review aimed to discuss the current research on the effect of doulas on maternal and fetal outcomes.

## Review

Methods

Eligibility Criteria

Articles that were peer-reviewed, written in English, and based on research studies conducted in more economically developed countries (MEDCs) between January 1, 2000, and December 31, 2021, were included. Other inclusion criteria included birth outcomes of pregnant women delivering in hospitals or homes with and without professional support personnel. Randomized controlled studies, case studies, case series, and cross-sectional studies were included. Knowledge syntheses such as systematic reviews and meta-analysis literature reviews were excluded. This scoping review was limited to studies conducted in MEDCs as medical and peripartum care differs significantly between economies. This review was also limited to studies with women younger than 35 years of age who were not high-risk pregnancies and who did not give birth incarcerated to limit any confounding variables. Although this review focused on doula support in standardized patient populations, women who used alternative methods of delivery in conjunction with or without doula support or standardized medical practice were also excluded. Moreover, this review focused on the peripartum period, specifically, labor and delivery.

The use of the term “continuous support” during the peripartum period in this review implies personnel outside a pregnant woman’s network, whose sole duty is to supply support during the laboring process. The use of continuous support only by guided professional medical practitioners within a hospital or clinical care settings without doula support is outside the scope of this review and only used within reviewed studies as controlled variables for comparison to persons using both resources and solely doula support alone.

Information Sources

The research team worked together to create a search strategy. Due to the specificity of the topic, the base search strategy was constructed using the key terms “doulas” and “labor outcomes” via a preliminary review of relevant articles in the PubMed and EBSCOhost databases. The following databases were searched: PubMed and EBSCOhost, using these key terms. The search was conducted in October 2022, and the base search was adapted to each database.

**Figure 1:**
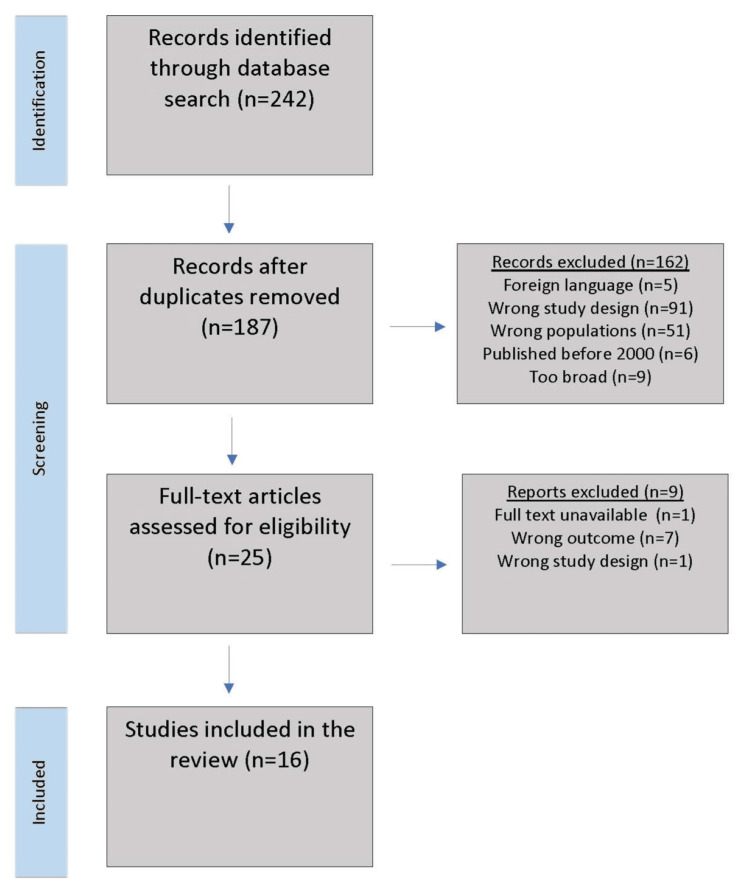
PRISMA flow diagram PRISMA: Preferred Reporting Items for Systematic Reviews and Meta-Analysis

The initial search identified a total of 242 citations. First, 61 duplicates were removed, leaving 187 studies to be screened. The research team reviewed titles and abstracts and reached an individual decision about articles for further consideration. Team members screened those articles for inclusion and exclusion criteria, as the search strategy section specified. Discussions ensued between the first three authors until agreement occurred, leaving 16 studies retained for the final review. Figure 1 depicts the article search strategy and selection process.

Search Strategy

The review question was “How does doula support impact birth outcomes?” Based on this question, data collection was specifically focused on the availability of articles on doulas in general but eventually further narrowed down to articles that also specifically mentioned birth outcomes and labor support. Due to the limited number of articles on the topic, Boolean operators and keywords were matched and used alongside each other to find all available articles that even slightly corresponded with the research topic. A substantial portion of the data included reflections or personal blogs of mothers who used doulas. Included were primary studies that had a healthy patient population and were conducted in MEDCs between 2000 and 2022 and investigated how doulas contributed to birth outcomes.

This review specifically chose doulas and not midwives or other delivery support because doulas receive their own unique training, and this way, it would be possible to streamline the outcomes of this review to potentially support doula training programs. The birth outcomes this review focused on were short-term, for example, delivery method, lactation, tearing, and Apgar score. Studies that focused on longer-term outcomes such as the development of the child over time or parenting style were not included.

Critical Appraisal of the Evidence

The Joanna Briggs Institute (JBI) Appraisal Tools was used to assess the risk of bias as well as the quality of research articles by categorizing them into high, moderate, and minimal risk bias. JBI uses checklists that categorize articles by type of study and strength of evidence. Using JBI appraisal tools, it was possible to evaluate the quality of the selected articles and review any potential biases they might have. Using the critical appraisal instrument suitable for each study type, it was concluded that all 16 articles had minimal risk of bias and were considered of good quality.

Results

Overview

The effect of doula support during peripartum was discussed in this review using 16 primary studies. Doula care in perinatal care was significantly correlated with positive delivery outcomes including reduced cesarean and premature deliveries. Doula support, specifically in low-income women, was shown to improve breastfeeding success, with quicker lactogenesis and continued breastfeeding weeks after childbirth. The emotional support provided by doulas was seen to reduce anxiety and stress during the labor period and reduce the length of labor. Women with a doula during childbirth helped raise their confidence and autonomy throughout labor.

Doula-Based Delivery Support

Undergoing labor can be a high-risk medical circumstance with potential complications. Cesarean deliveries mitigate a variety of those potential complications but come with their own risk and detriment to the patient. The usage of doulas through the labor process decreases the use of cesarean deliveries, thus improving the probability of positive labor outcomes for the pregnant patient and the baby. The Dona International Data Project (DIDP) observed 1,892 mothers under the care of 574 doulas over a 13-year period, of which 52.7% were referred by the patient’s medical team despite 64.7% of the mothers already seeing an obstetrician [22]. This shows a large-scale institutional backing of third-party care providers for obstetrical support. The study also found improved outcomes and lower rates of intervention relative to national statistics across their entire sample population, of which 14% were labeled high-risk pregnancies [22]. This was referenced by a 7.8% drop in cesarean surgery (12.6% versus 20.4% nationally) and a 5.01% drop in prematurity (4.9% versus 9.91% nationally), noting a statistically significant correlation between positive delivery outcomes with doula support. Although low birth weight (LBW) deliveries across the entire sample population of mothers were higher than the national standard at 10.2% (up from 9.48% nationally), there was a significant drop to just 7% when separately accounting for the 90.03% of total mothers who were delivering their first child [22]. This metric falls below the Healthy People 2020 target for LBW rate at 7.8%, demonstrating the efficacy of doula-based delivery to decrease cesarean delivery prevalence [22].

One study that analyzed data from the Birth Companions Program at the Johns Hopkins University School of Nursing further reinforces the validity of doula services decreasing cesarean delivery utilization through their assessment of a program where nursing students trained as doulas to provide interventions [23]. The role of birth companions and their impact was compared with certified nursing midwives and obstetricians. When comparing these groups and their outcomes, it was found that birth companions provided about one more physical intervention (ambulation, position changes, etc.) per birth than obstetrician clients [23]. Birth companions provided verbal encouragement to their clients and eased their client’s fears. Furthermore, it was found that birth companions were more likely to report answering clients’ questions when compared to certified nursing midwives [23]. With an increase in the total number of interventions, there was a decrease in the odds of epidural use and cesarean birth; however, emotional interventions did not have this impact. Emotional interventions not exerting the same statistically significant impact as doula inventions on cesarean incidence rates reinforce the need for the delineation between the two types of delivery support systems. In a similar study, doula support was more thoroughly defined. The study cited doula support as close physical proximity, touch, and eye contact with the laboring woman and teaching, reassurance, and encouragement of the woman and her male partner [24]. Results showed that for a middle-class woman with a male partner and doula support, the cesarean rate was 13.4% compared to the control group at 25%. Similarly, in those with induced labor, women with doula support had a cesarean rate of 12.5% compared to the control at 58.8% [24]. The use of well-defined doula support proves to be a reliable method to decrease the usage of cesarean deliveries and, thus, improve patient delivery outcomes by preventing the accompanying risks and complications of cesarean deliveries.

Efficacy of Doula Support

The use of epidurals during delivery is an indication of how well the pregnant patient’s pain is managed, which is a direct correlation to maternal well-being throughout the process. One study determined that doula-supported mothers were observed to be more likely to attend childbirth preparation classes (50% versus 10%), less likely to use epidural/pain medication during labor (72% versus 83%), more likely to initiate breastfeeding (81% versus 74%), and more likely to utilize car seats at three weeks postpartum (97% versus 93%). These studies showed that doula care may affect both child and maternal health and safety during and after childbirth [25].

The initiation and continuity of breastfeeding are verified predictors of neonatal and infant health outcomes. One study assessed the effect of doula care on breastfeeding outcomes in low-income women who were giving birth for the first time [26]. Doula care was associated with a shorter stage 2 of labor and an earlier onset of lactogenesis, within 72 hours postpartum [26]. The women that received doula care were also more likely to be breastfeeding at six weeks. This suggests that doula intervention throughout the labor process improves not only the maternal labor outcomes but also the health outcomes and well-being of the infant through the continued support and education of the new mother.

Efficacy of Doula Labor Interventions for Decreasing Infant Mortality

The support and benefits that doulas provide women during pregnancy and childbirth have been associated with a positive impact on women’s emotional well-being and overall satisfaction. Access to the services a doula can provide is sometimes limited to women due to extrinsic factors, such as a lack of information about the services and the cost of services. The By My Side Birth Support Program (BMS) was established to provide doula support to women living in neighborhoods with a high burden of poor health, specifically a high infant mortality rate. BMS provides support during pregnancy, delivery, and the postnatal period. One study assessed the effect of the BMS program on birth outcomes and each woman’s overall experience with the doula program [27]. Follow-up phone interviews found that 95.9% of women would recommend the BMS program or use it in a future pregnancy [27]. BMS participants had lower rates of preterm birth and low birth weight; however, the cesarean section rate was statistically similar [27]. However, it was not possible to determine if there was a causal relationship between BMS program exposure and birth outcomes because of the self-selection of the participants and other possible sources of bias.

Doulas as Maternal Mental Well-Being Support System

Maternal mental health is a crucial part of a holistic definition of health and a consistent predictor of both delivery and perinatal outcomes for both mother and infant. Childbirth can be a traumatic event for both mother and child, and preterm anxiety can cause an increase in acute stress directly following birth and possible post-traumatic stress disorder (PTSD). One study explored doula care and its role in alleviating pre-term trait anxiety. The study followed 149 women by measuring prenatal trait anxiety at the end of pregnancy, acute stress at two days postpartum, and symptoms of PTSD one month postpartum [19]. Preliminary results showed that doula support during the prenatal period may reduce the anxiety-related risk of developing post-traumatic stress disorder following childbirth [19]. The dynamic support of doulas throughout the perinatal and postnatal periods can decrease the mental health morbidities of the birth process and establish a mental health support system, enabling new mothers to be within a mental and emotional safety net.

Interviews of 137 low socioeconomic English women, of whom 75 (54.7%) experienced doula support, highlighted the impactful social bonds fostered by doulas in addition to the healthcare they provided [28]. Mothers often reported distress upon separation from their doulas on whom they previously depended for social support. This suggests that doulas may play just as important a role postpartum as they do before and during delivery [28]. Overall, most women reported that the doula service had positive impacts on their emotional well-being. The doula service provided women with someone to listen to their concerns as well as gain confidence and belief in themselves during delivery. One important finding was that these benefits did not depend on the timing of the help, whether it was prior, during, or after birth [28]. Women also found the doula service to help strengthen their relationship with their partners. The women viewed the doula volunteer as a family member or friend and valued their one-on-one relationship, allowing a feeling of support, regardless of their current social network [28].

Doula-Based Interventions to Decrease Disparities

Women of a lower socioeconomic status (SES) have decreased maternal and infant health outcomes compared to their higher SES peers. Doula interventions throughout labor can serve to close the gap between these outcomes and alleviate the burden of health disparities for lower SES women. A recent retrospective cohort study sampling 298 women receiving Medicaid with doula support across three states in the USA found an overall 52.9% decrease in the risk of cesarean surgery and a 57.5% decrease in rates of postpartum depression/postpartum anxiety (PPD/PPA) versus their counterparts without third-party supportive care [18]. Mothers who received doula care solely during delivery saw an even greater, 64.7%, decrease in PPD/PPA, highlighting the potential value of such care during a relatively short but critical period [18].

One study assessed pregnancy-related outcomes of a program called Embrace Refugee Birth Support, which provides a pregnancy support program to refugees [29]. Pregnancy-related outcomes were compared to a group of women from the same community and racial/ethnic background. Participants of the Embrace Refugee Birth Support program had better pregnancy-related outcomes, such as higher gestational age and reaching full term [29]. There were also lower odds of cesarean delivery and low birth weight [29].

Continuous labor support within lower SES communities is shown to increase positive outcomes with vaginal births as well as cesarean births. A study showed that women with doula support had lower rates of non-indicated cesareans compared to those who wanted but did not have doula support and those that did not desire doula support [30]. Black women, women on public health insurance (Medicaid and other government-funded programs), and women without health insurance were shown to have higher rates of adverse birth outcomes when compared to women with private health insurance [30]. Expanding access to doula care, especially to those who desire doula care but did not receive it, would help facilitate lower rates of non-indicated cesareans. In addition, doulas in low socioeconomic areas resulted in near-universal breastfeeding initiation at 97.9%, compared to 80.8% of those in the general Medicaid population [16]. Additionally, the study found that 92.7% of African American mothers receiving doula support initiated breastfeeding compared with 70.3% of the general Medicaid population [16]. Doula support throughout perinatal and postnatal periods was found to be an effective method to overcome the social determinants of health [16].

Another study exploring the impact of doulas on healthy birth outcomes divided 226 expectant and socially disadvantaged mothers at risk for adverse birth outcomes into two groups [31]. One group was given pre-birth assistance from a certified doula, while the other group participants did not [31]. The results of the study showed that the expectant mothers matched with a doula had better birth outcomes, were four times less likely to have a low-birth-weight baby, were two times less likely to experience a birth complication, and were significantly more likely to initiate breastfeeding [31]. A comprehensive system of psychosocial and health support was thought to be the main reason for the improvement in birth outcomes [31].

Cost-Effectiveness of Doula Support

Formal doula support throughout the prenatal, perinatal, and postnatal periods of pregnancy can increase the probability of positive labor outcomes. In an urban and multicultural cohort of 11,471 women within a hospital-based doula support program with births at 37 weeks or greater, women who received doula support expressed lower rates of cesarean deliveries and significantly higher rates of breastfeeding intent and early initiation, regardless of parity or provider except for multiparous women with physician providers [32]. However, the benefits of doula support may be generalized beyond full-time hospital-based doulas. A group of 600 low-risk, nulliparous women enrolled in a randomized clinical trial to determine the benefit of a low-cost alternative to professional doulas. Within this cohort, labor support was provided to the pregnant patient by a minimally trained female friend or relative, selected by the mother-to-be. These support figures attended two two-hour classes about providing non-medical, continuous support to laboring women, with the pregnant patient. Compared to mothers who received standard care, the women with a lay doula had decreased labor times, increased Apgar scores at one and five minutes, and a downward trend with cesareans [33]. In a secondary study, where researchers followed up with the mothers 6-8 weeks postpartum, they found that these doula alternative-supported mothers had increased reports of positive prenatal expectations about childbirth, positive perceptions of their infants, increased self-worth, and increased support from others [34]. In addition, the doula alternative-supported mothers also reported overall satisfaction with their hospital-based care and increased probability of breastfeeding [34]. Table 1 summarizes the primary research articles included in this review.

**Table 1:** Summary table of the 16 articles in the review PTS-FC: post-traumatic stress following childbirth, AS-FC: acute stress immediately following childbirth

Authors	Purpose	Study design	Study population	Methods	Limitations	Key findings
Campbell et al. (2007) [34]	To investigate the association between support with a doula and the perception by the mother of herself, the infant, and the support of others at 6-8 weeks postpartum	Randomized controlled trial	600 low-risk, nulliparous women	300 with doula support (minimally trained family member or friend), 300 with standard care, and 494 participants completed a second study over the phone of a 42-item questionnaire	There was a significant difference between the groups regarding race, there was no blinding for the research assistant conducting the interviews, recall bias could have affected the responses, and there was a lack of standardized measures of validity and reliability utilized in the study	Doula support by a minimally trained female selected by the mother was an inexpensive way to improve the outcome of both mother and infant.
Mottl-Santiago et al. (2008) [32]	To determine whether hospital-based doula support during labor influenced birth and breastfeeding outcomes	Retrospective cohort study	11,471 full-term women (greater than or equal to 37 weeks gestation) during labor	Tested over the first seven years of the hospital-based doula program, full-term women (greater than or equal to 37 weeks gestation) with and without doula support during labor, regression analyses were used to compare outcomes	Possible experimental group bias utilized a preexisting database, which prevented researchers from collecting certain variables, and limited demographic analysis in regards to distinguishing ethnic group and racial categories	The hospital-based doula support program used during labor had significantly higher breastfeeding rates than those who did not have doula support.
McGrath et al. (2008) [24]	To investigate how nulliparous women with a middle income and a male partner present during labor and birth were perinatally affected by doula support	Randomized controlled trial	420 nulliparous (never given birth), low-risk women in the third trimester	224 women with a doula present from admission through labor and delivery, 196 women without a doula	None noted	The presence during labor of a doula significantly decreased the need for an epidural and the possibility of cesarean delivery, and all women and their partners had positive opinions regarding the presence of a doula during labor and delivery.
Kozhimannil et al. (2014) [30]	To identify women who were able to use doula support and those that did not but desired to use doula support and determine the relationship between the desire of doula services, utilizing doula services, and cesarean delivery with evaluation of non-indicated cesareans	Retrospective cohort study	2,400 women 18-45 who delivered a single infant in the USA from 2011 to 2012	Listening to Mothers III Survey given to women who were a part of Harris Interactive maintained online panels	Self-reported study, Listening to Mothers Survey did not have clinical or diagnostic data due to its self-reporting nature, and a limited sample size	Women with doula support compared to those without doula support and those without support but desired it had lower rates of cesarean delivery overall and non-indicated cesarean delivery.
Everson et al. (2018) [22]	To examine a nationwide sample of the outcomes of both adolescent mothers and neonates who received doula support during birth from 2000 to 2013	Retrospective cohort study	1,892 adolescent mothers (included 1,896 infants, four sets of twins) in the USA between 2000 and 2013 that had doula support present	DONA International birth doula collection form was filled out by the doula and mother; entries included those of adolescent mothers (ages 15-19) between 2000 and 2013	The collection form was not a research design so important covariables were not collected, data entry errors could have occurred as the information was input twice (once by the doula and again by volunteers entering data into the Master file), possible undefined confounding variable of midwifery-led care, and true outcomes need to be gained by a study including a comparison group	The adolescent mothers and their infants in doula-supported births had lower intervention rates and better health outcomes than the national statistics for adolescent births.
Kozhimannil et al. (2013) [16]	To compare the effect on birth outcomes in Medicaid recipients of prenatal education and delivery support from doulas to a national sample to determine cost-saving potential	Retrospective cohort study	279,008 nationwide singleton births on Medicaid, 1,079 singleton births on Medicaid that were doula-supported in Minnesota	Analyzed Medicaid-funded births nationwide and those in Minnesota that were doula-supported using descriptive statistics to determine the effects of doula care and cost deduction associated with decreased cesarean delivery	The doula care only included one state, the nationwide sample and the doula sample were collected during two different periods, the information was collected from discharge reports that could have included under-reporting compilations of doula-supported births, possible selection bias in those Medicaid beneficiaries who chose doula-supported care, and the cost of delivery was estimated	Due to the decreased rate of cesarean deliveries in doula-supported birth and as a result decreased cost of delivery, Medicaid programs should consider covering doula services for birth.
Gruber et al. (2013) [31]	To compare birth outcomes in mothers with and without doula support that are enrolled in the same education program	Prospective cohort study	226 pregnant women enrolled in a healthy moms and healthy babies’ childbirth education class between January 2008 and December 2010	Mothers who attended at least three of the classes were given the choice to work with a doula from the Healthy Beginnings Doula Program, 129 pregnant women without doula support, 97 pregnant women with doula support	Participants self-selected to work with a doula, no information was provided on the mother’s other support systems outside of the doula, and the doula support was not the only service and support provided to the mothers	Mothers receiving doula support had better birth outcomes when compared to those without doula support in regard to low birth weight, complications experienced during birth and delivery, and breastfeeding initiation.
Falconi et al. (2022) [18]	To determine when doula care initiation and with which clinical providers will have the best effect on expectant mothers and determine whether there is a difference in outcomes with a doula based on health status and race/ethnicity	Retrospective cohort study	596 women (298 pairs of women that were matched on age, hospital type, state, socioeconomic status, and race/ethnicity where one received doula support and the other did not)	Collected Medicaid claims from multiple states in the USA between January 1, 2014, and December 31, 2020, to compare the birth outcomes of those women that did and did not have doula support	The extent of doula care was not evaluated, the population of women could not be generalized to the broader US population, and the concordance of race between the doula and the mother was not addressed in its possible influence on health outcomes	Women receiving doula support had lower rates of cesarean delivery and postpartum depression/anxiety diagnosis compared to those without doula support
Hans et al. (2018) [25]	To investigate the effect of doula’s performing home visits on newborn care practices, birth outcomes, and postpartum health of both mother and infant	Randomized controlled trial	312 young pregnant women	Women randomly assigned to doula home services (experimental) or case management services (control), interviews created a baseline then at 37 weeks, three weeks postpartum, and three months postpartum, the participants completed the interview again	The small sample population included only four home visit doula programs in one state, high-risk adolescent women were excluded, and the information was provided by the mother so information on administrative records was not available	Compared to the case management group, the doula home visit group was more likely to attend prenatal education courses, less likely to utilize epidural or other pain medication in labor, and had a higher likelihood of initiating breastfeeding with their infant
Darwin et al. (2017) [28]	To evaluate trained volunteer doula services for disadvantaged women in England	Program evaluation	137 women who received a volunteer doula service before December 2012	Questionnaires (n=136) were done with the help of a researcher or interpreter or were self-completed, individual or group interviews (n=12) were used also	Low response rate of the questionnaire (21.7% of women who used the service) and recipients of the survey could not be contacted from many years prior	Women with the volunteer doula service reported benefits in emotional health and well-being. Most women also viewed the volunteers as a constant help, focused on the mother throughout labor and delivery. An important finding was that the benefits of the volunteer did not depend on the timing of the help, whether it was prior, during, or after birth.
Paterno et al. (2012) [23]	To evaluate a student-nurse doula program specifically in interventions, labor analgesia, and cesarean birth	Secondary analysis	648 records of mothers in the Birth Companions Program	T-tests, chi-squared statistics, and logistic regression models	The data depended on the birth companion’s documentation; the length of labor could not be used as a variable	Epidural use and cesarean birth occurrence decreased with an increase in the number of interventions used by birth companions.
Thomas et al. (2017) [27]	To evaluate the New York City Department of Health and Mental Hygiene’s By My Side Birth Support Program (BMS)	Program evaluation	489 mothers’ data collected by doulas who took part in the By My Side program from 2010 to 2015	BMS analyzed the demographic characteristics, birth outcomes, and follow-up interviews of the women they served from 2010 to 2015	Self-selection bias	The BMS program provided benefits to disadvantaged communities. BMS participants had lower rates of preterm births and low birth weight infants. In follow-up interviews, program participants stated that they would recommend the program to others.
Nommsen-Rivers et al. (2009) [26]	To examine associations between doula care, early breastfeeding outcomes, and breastfeeding duration	Prospective cohort study	Low-income, full gestation primipara receiving doula care (n=44) or standard care (n=97)	The authors collected data from hospital records on birth outcomes and feeding data, follow-up interviews were conducted for lactogenesis onset and breastfeeding behavior	Selection bias, certain results were gained from maternal recall, lack of population generalizability, and the Doula Care Project had a narrow range of eligibility criteria	Doula care resulted in a shorter stage 2 of labor and the onset of lactogenesis within 72 hours postpartum. Doula care patients were 89% more likely to start breastfeeding by six weeks when compared to standard care patients.
Mosley et al. (2021) [29]	To evaluate birth outcomes of a community of refugee women in Georgia	Program evaluation	9,136 hospital clinical records of mothers taking part in the Embrace Refugee Birth Support program from 2016 to 2018	They used bivariate tests to compare the Embrace participants and the comparison group’s descriptive statistics, they used chi-squared tests for categorical predictors and outcomes and T-tests for continuous predictors and outcomes, they also used multivariate analyses	No data on the refugee status of women in the comparison group	The Embrace participants had a 48% lower chance of labor induction when compared to the comparison group. Embrace participants were also less likely to have cesarean deliveries.
Campbell et al. (2006) [33]	To compare labor outcomes between women with doula support and women with no additional support	Randomized controlled trial	600 nulliparous (never given birth) women	They had a control group who were women who did not have doula support and an experimental group who had doula support, the outcome measures were the length of labor, type of delivery, type and timing of analgesia/anesthesia, and Apgar scores	No statistical significance for the type and timing of analgesia/anesthesia and the type of delivery	The length of labor was statistically shorter in the doula support group. The doula group also had higher Apgar scores.
Rousseau et al. (2021) [19]	To determine the risk of developing PTS-FC in women who had prenatal anxiety and if doula support had any effect on PTS-FC development	Longitudinal cohort study	149 low-risk nulliparous pregnant women	The women were split into a doula group and a non-doula group, the mothers self-reported via questionnaires during the last trimester (State-Trait Anxiety Inventory-trait questionnaire), about 48 hours after delivery (Stanford Acute Stress Reaction Questionnaire), and one month postpartum (Posttraumatic Stress Disorder Checklist- Specific Version), the analysis was run on the group to determine PTS-FC development and then compared the doula and non-doula groups to determine the effect of doula support	Analysis of the doula support was secondary instead of primary; doula care itself was not analyzed; it was not a randomized controlled design, which could influence results based on mothers’ feelings toward doulas; small doula group; no clinically diagnostic interviews of the mothers to determine prenatal trait anxiety, AS-FC, and PTS-FC; possible lack of generalizability based on exclusion criteria; and lack of investigation of other vulnerability factors such as state anxiety that could contribute to PTS-FC or AS-FC development	Prenatal trait anxiety and AS-FC are significant risk factors for PTS-FC. Doula support was found to be a possible mitigating factor for PTS-FC development.

Discussion

The purpose of this scoping review was to examine the benefits of doula support in labor outcomes. The impact of doula support in an array of perinatal populations including underrepresented and underserved women was explored. Of the findings in this review, decreased incidence of cesarean, premature labor, and low birth weight was the most remarkable. Some possible explanations may include improved prenatal education on birth expectations and increased empowerment and autonomy during birth [31]. Higher birth weight and lowered rates of cesarean could also be due to reduced induction rates in women with doula support. Epidural and medical pain management was also reduced among women with doula support. This could be due to increased childbirth preparedness prior to birth, increased positional flexibility during labor, and coping strategies provided by doulas during labor [24].

Overall, it was found that doula support can be beneficial as it provides emotional and physical support during parturition [23]. The results indicated that there are reduced infant mortality rates and epidural use, as well as increased lactogenesis in patient populations utilizing doulas [25,27]. Furthermore, doula intervention and physical support could result in decreased monetary expenses from fewer procedures performed and an increased incidence of breastfeeding, with the doula support itself coming from cost-effective programs [23]. Physical benefits include but are not limited to breathing techniques, changing positions of the mother during labor, and linking physician-patient communication. Improved mental health status, involving elements of reduced rates of anxiety, depression, and postpartum PTSD, were social and emotional benefits provided by doulas [19]. Studies that clearly defined doula support were better able to demonstrate the positive impacts of having a doula than studies with a looser definition [24]. This indicates the need for future studies to have a solid definition for their doula interventions. Some studies compared the difference in labor outcomes of mothers who were offered doula support to those who were offered it and did not utilize it [16]. However, most studies just compared the mothers who had the presence of a doula during labor to the mothers that did not. In this case, an area of investigation is needed to see if any comparisons were made between labor outcomes in mothers who knew of doula support and used it to the outcomes of mothers who had no knowledge of doula support programs and did not use it. It thus raises the question of whether the mothers who knew of doula support had the knowledge and utilization of other services that may have contributed to positive results of doulas. A more focused definition of doula support outlining the scope of education and knowledge that the mother will receive that is exclusively offered only by doulas will further strengthen the argument that doula support directly correlates to the positive labor outcomes shown. A precise intervention definition would also allow a better translation to doula training programs. A defined interventional role would provide consistency of training and help to ensure uniform support for pregnant women and their children.

While this review focused on short-term birth outcomes, there is reason to believe that doula support would have long-term benefits. Studies included in this review found that doulas increased breastfeeding rates, which can have long-term positive impacts on both the mother and child. Studies also found that doula support affected car seat utilization [25] and improved the postnatal mental health of the mother [34]. Both outcomes show the role of doula support past the labor itself and highlight an area where further investigation may be warranted. It would also be beneficial to continue the investigation with the separation of the time that the doula is used such as before labor, during parturition, and postpartum. Therefore, each specific labor outcome could be connected to the specific doula interventions used to continue to promote and encourage the training of those interventions in doula training programs. It would also give a better understanding of the proper time to start doula support to fully maximize its benefits.

An interesting finding was a significant decrease in cesarean rates among Medicaid-funded births in patients with doula support as opposed to those without. While the cost of doulas may come with an additional cost, utilization may reduce the cost of labor by decreasing the rates of adverse events during childbirth [16]. This raises the question as to whether doula support may improve outcomes and lower health disparities among mothers from a lower socioeconomic status. Both a natural birth and the ability to breastfeed not only have short- and long-term benefits for mother and child but are cost-effective as well. Implementing doulas in public health insurance and for lower-income mothers is a straightforward way to overcome a major issue in healthcare today. This could be made possible with increased doula training programs that can be offered to not only healthcare professionals but also a friend or family member of the birthing mother [34]. Using studies such as this one, doula training can be improved and made more accessible.

Limitations of the Review Process

In this scoping review, only studies published in English were included, which could bias the results toward English-speaking countries and exclude any studies done in non-English-speaking countries. Additionally, only studies performed in MEDCs were included because the purpose of the study was to evaluate the benefits of using a doula in an otherwise uncomplicated and modern healthcare setting. Using a doula in a developing country healthcare setting likely has its own set of benefits that were not investigated in this review.

Limitations of the Studies Used for the Review 

Several of the studies included surveys or self-reported data. These research methods can leave room for a bias of women who feel comfortable participating in reporting outcomes, and the results may not be generalizable. None of the studies included were blinded studies as it would not be possible to blind a laboring woman to have the care of a doula. Moreover, some of the included studies had small sample sizes, which also could limit the generalizability of the results, and the findings must be taken with caution.

## Conclusions

Labor and delivery are events that can present a high medical risk to mothers and their children. Doulas can ease this process in a multifactorial capacity, but it is important first to properly define their role. With a clear definition, the responsibilities of the doula can be filled adequately by thoroughly trained individuals. This could help ensure that the mother and child will reap the potential benefits of having a doula. This scoping review presented data that support the presence of a doula; doulas have been found to reduce cesarean section frequency, low birth weight, and premature labor. Evidence shows that decreased cesarean sections have led to better outcomes for the mother and the child. Doula intervention has also been correlated with a decrease in epidural use during delivery, increased rates of breastfeeding, and the use of safety precautions for the child. The advocacy that doulas provide can increase well-being and satisfaction concerning the birthing process, and it provides education and support. This support may reduce mental health morbidities, such as PTSD, in mothers without a support system. The implementation of doulas as a common healthcare entity could be helpful, particularly for women who experience healthcare disparity. Access to doulas can be limited due to a lack of information as well as financial constraints. Future efforts should be focused on promoting the use of doulas and researching their positive impact. More research would help define their roles in more complicated healthcare environments than the ones addressed in this scoping review and identify the positive or negative implications of their presence. Doulas can be a means by which better healthcare is provided to mothers and their children during parturition.
