# Correction: The Effect of Doulas on Maternal and Birth Outcomes: A Scoping Review

**DOI:** 10.7759/cureus.c310

**Published:** 2025-09-23

**Authors:** Alexandria Sobczak, Lauren Taylor, Sydney Solomon, Jodi Ho, Scotland Kemper, Brandon Phillips, Kailey Jacobson, Courteney Castellano, Ashley Ring, Brianna Castellano, Robin J Jacobs

**Affiliations:** 1 Medicine, Nova Southeastern University Dr. Kiran C. Patel College of Osteopathic Medicine, Fort Lauderdale, USA

This article has been corrected to fix the following typos in the PRISMA diagram and preceding paragraph:

- Duplicates removed: 58 corrected to 61
- Records after duplicates removed: 184 corrected to 187
- Records excluded: 159 corrected to 162

